# Molecular Mechanisms in the Carcinogenesis of Oral Squamous Cell Carcinoma: A Literature Review

**DOI:** 10.3390/biom15050621

**Published:** 2025-04-25

**Authors:** Laertty Garcia de Sousa Cabral, Isabela Mancini Martins, Ellen Paim de Abreu Paulo, Karina Torres Pomini, Jean-Luc Poyet, Durvanei Augusto Maria

**Affiliations:** 1 Faculty of Medicine, University of Sao Paulo (FMUSP), Sao Paulo 05508-220, SP, Brazil; laertty.c@usp.br (L.G.d.S.C.); ellen.paulo.esib@esib.butantan.gov.br (E.P.d.A.P.); 2 Laboratory of Development and Innovation, Butantan Institute, Sao Paulo 05585-000, SP, Brazil; i.martins.proppg@proppg.butantan.gov.br; 3 Department of Biochemistry and Pharmacology, School of Medicine, University of Marília (UNIMAR), Marília 17525-902, SP, Brazil; karinatorrespomini@unimar.br; 4 INSERM UMRS1342—CNRS EMR8000, Institut De Recherche Saint-Louis, Hôpital Saint-Louis, 75010 Paris, France; 5 Université Paris Cité, 75006 Paris, France

**Keywords:** oral squamous cell carcinoma, tumor microenvironment, targeted therapies

## Abstract

The tumor microenvironment (TME) plays a crucial role in the development, progression, and metastasis of oral squamous cell carcinoma (OSCC). The TME comprises various cellular and acellular components, including immune cells, stromal cells, cytokines, extracellular matrix, and the oral microbiome, all of which dynamically interact with tumor cells to influence their behavior. Immunosuppression is a key feature of the OSCC TME, with regulatory T cells (Tregs), myeloid-derived suppressor cells (MDSCs), and tumor-associated macrophages (TAMs) contributing to an environment that allows tumor cells to evade immune surveillance and supports angiogenesis. The oral microbiome also plays a pivotal role in OSCC pathogenesis, as dysbiosis, or imbalances in the microbiota, can lead to chronic inflammation, which promotes carcinogenesis through the production of pro-inflammatory cytokines and reactive oxygen species (ROS). Pathogens like *Porphyromonas gingivalis* and *Fusobacterium nucleatum* have, hence, been implicated in OSCC-driven tumor progression, as they induce inflammation, activate oncogenic pathways, and modulate immune responses. In this review, we discuss how the interplay between immunosuppression and microbiome-driven inflammation creates a tumor-promoting environment in OSCC, leading to treatment resistance and poor patient outcomes, and explore the potential therapeutic implication of a better understanding of OSCC etiology and molecular changes.

## 1. Introduction

OSCC is one of the most common types of head and neck cancer, accounting for approximately 90% of cases in this anatomical region [1,2]. This type of carcinoma is characterized by the uncontrolled growth of the squamous epithelial cells of the oral cavity, resulting in lesions that can lead to disfiguration and functional disability as well as significant mortality [3,4]. The incidence rate of OSCC varies globally, with higher prevalence observed in regions such as Southeast Asia, where factors like tobacco and betel use are strongly associated with the development of the pathology [5,6].

The underlying molecular mechanisms of OSCC carcinogenesis are complex and involve a series of genetic and epigenetic alterations that contribute to the malignant transformation of oral mucosa cells [7]. The emergence of gain-of-function p53-mutant oncogenes or mutations in oncogenes such as *RAS*, as well as the inactivation of tumor suppressor proteins like p16INK4a, are common events in the progression of this type of carcinoma [8]. Additionally, genomic instability, resulting from mutations and aneuploidy, plays a crucial role in promoting tumor heterogeneity and treatment resistance [9].

The carcinogenesis of OSCC is also closely linked to cellular alterations that favor the uncontrolled proliferation and apoptosis evasion of OSCC cells [10]. Signaling pathways such as the epidermal growth factor receptor (EGFR) and the PI3K/AKT pathways are also often dysregulated, promoting OSCC cancer cell growth and survival [11,12,13]. Angiogenesis, mediated by factors like vascular endothelial growth factor (VEGF), facilitates tumor nutrition and metastatic spread [14,15].

**Figure 1:**
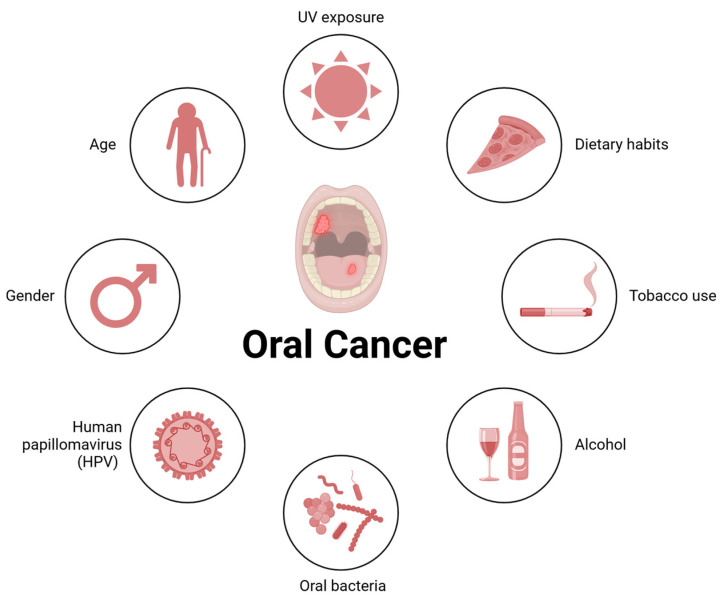
Risk factors for oral cancer (OSCC). Risk factors include UV exposure, tobacco use, alcohol consumption, dietary habits, and oral bacteria. Additionally, demographic factors such as age and gender, along with infections with high-risk human papillomavirus (HPV), particularly HPV-16, have been associated with the etiology of this cancer.

Importantly, environmental and behavioral risk factors (Figure 1), such as smoking and alcohol consumption, are well documented in the literature as significant contributors to OSCC development [16]. Furthermore, infections by human papillomavirus (HPV), especially high-risk subtypes like HPV-16, have also been implicated in OSCC etiology, emphasizing the need for targeted prevention strategies [17,18].

Understanding the molecular and cellular mechanisms involved in OSCC carcinogenesis is crucial for the development of more effective therapies [19]. Current therapeutic approaches, which include the use of tyrosine kinase inhibitors and immunotherapies, have shown promising results in the treatment of this carcinoma [20]. However, treatment resistance and tumor heterogeneity remain challenging, highlighting the need for new therapeutic approaches [20].

This review aims to provide a comprehensive and updated overview of the molecular mechanisms involved in the carcinogenesis of OSCC, highlighting the main genetic and epigenetic alterations, deregulated signaling pathways, and environmental and behavioral risk factors as well as their therapeutic implications.

## 2. Methods

This systematic review was conducted following the guidelines of the Preferred Reporting Items for Systematic Reviews and Meta-Analyses (PRISMA). The aim was to compile and synthesize available evidence concerning the molecular mechanisms in the carcinogenesis of OSCC.

### 2.1. Search Strategy

A comprehensive literature search was conducted across the PubMed, Scopus, Web of Science, and EMBASE databases, covering the period from January 2000 to February 2025. The search strategy used Boolean combinations of the following terms: “oral squamous cell carcinoma”, “OSCC”, “molecular mechanisms”, “pathogenesis”, “therapeutic approaches”, “treatment resistance”, “molecular targets”, “immune evasion”, and “risk factors”. Search strings were adapted for each database to ensure optimal retrieval.

### 2.2. Eligibility Criteria

Studies were included if they met the following criteria: (a) full-text availability; (b) publication in English; and (c) direct relevance to the molecular pathogenesis, therapeutic targets, or mechanisms of resistance associated with oral squamous cell carcinoma. Studies that focused exclusively on other head and neck cancers, editorials, commentaries, letters, or duplicates were excluded. Additionally, studies that discussed OSCC only tangentially, without addressing its biological or therapeutic dimensions in depth, were also excluded.

### 2.3. Literature Selection and Synthesis

All retrieved titles and abstracts were screened manually by two independent reviewers to assess relevance. Full-text articles were then reviewed for final inclusion. Disagreements were resolved by consensus. From each selected article, key information was extracted, including (i) type of molecular alteration or pathway discussed; (ii) study context (clinical, experimental, or theoretical); (iii) relation to OSCC prognosis, progression, or treatment; and (iv) emerging therapeutic implications.

No quantitative synthesis (meta-analysis) was performed due to the narrative and thematic nature of the review. Therefore, the included studies were not formally graded using scoring systems such as STROBE. Instead, studies were qualitatively grouped and discussed according to emergent thematic axes.

### 2.4. Thematic Grouping

Based on content relevance and frequency across the literature, the studies were organized into the following major categories:

- Genetic and epigenetic alterations in OSCC;
- The role of the tumor microenvironment and immune evasion;
- Behavioral and environmental risk factors;
- Exosome-mediated signaling;
- Resistance mechanisms to conventional and targeted therapies;
- Promising molecular targets and ongoing experimental therapies.

**Figure 2:**
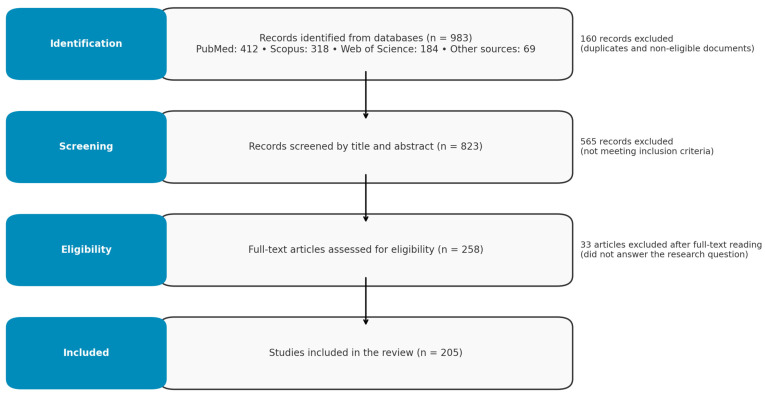
PPRISMA diagram of search and review process.

A total of 205 studies were included and analyzed within these categories. The full selection process is summarized in the PRISMA flow diagram (Figure 2).

## 3. Tumor Microenvironment (TME) in Oral Squamous Cell Carcinoma

The TME plays a pivotal role in the initiation, progression, and metastasis of OSCC [21]. It comprises a complex network of cellular and acellular components, including immune cells, stromal cells, extracellular matrix, cytokines, and microbiota, all of which interact dynamically with each other and with tumor cells, promoting cancer progression and resistance to therapy. In the context of oral cancer, the two main critical aspects of the TME that significantly impact OSCC development and progression are immunosuppression and the oral microbiome [21,22].

Immunosuppression within the TME is a hallmark of OSCC and contributes substantially to tumor evasion from immune surveillance, facilitating unchecked tumor growth and metastasis. Various mechanisms and cellular players orchestrate this immunosuppressive milieu [23,24]. A key contributor to immunosuppression in OSCC is the accumulation and activation of regulatory T cells (Tregs) [25]. Tregs suppress anti-tumor immune responses by inhibiting the activity of effector T cells and natural killer (NK) cells through the secretion of immunosuppressive cytokines such as interleukin-10 (IL-10) and transforming growth factor-beta (TGF-β) [26]. Elevated levels of Tregs in OSCC have been correlated with poor prognosis and reduced patient survival rates, underscoring their role in tumor progression [26,27]. Myeloid-derived suppressor cells (MDSCs) are another significant immunosuppressive cellular population within the OSCC TME. MDSCs inhibit T cell activation and promote the expansion of Tregs, further dampening immune responses against tumor cells. Mechanistically, MDSCs are involved in the production of arginase, nitric oxide, and ROS, which impair T cell function and proliferation. Furthermore, the presence of MDSCs has been associated with increased tumor invasiveness and resistance to conventional therapies in OSCC patients [28,29]. Tumor-associated macrophages (TAMs), particularly the M2-polarized subtype, also contribute to immunosuppression in OSCC. M2 macrophages secrete anti-inflammatory cytokines and growth factors that support tumor growth, angiogenesis, and metastasis while suppressing effective immune responses. Consequently, high densities of TAMs within OSCC tissues are linked to enhanced tumor aggressiveness and unfavorable clinical outcomes [30,31].

Additionally, OSCC cells often exploit immune checkpoint pathways to evade immune detection. The overexpression of programmed death-ligand 1 (PD-L1) on tumor cells, which interact with programmed death-1 (PD-1) receptors expressed on T cells, leads to T cell exhaustion and reduced cytotoxic activity [32]. This mechanism effectively blunts the immune system’s ability to engage and eliminate cancer cells. Of note, therapeutic strategies targeting the PD-1/PD-L1 axis have shown promise in restoring anti-tumor immunity and improving patient outcomes in OSCC [32,33].

### 3.1. Role of Exosomes in the Tumor Microenvironment of Oral Squamous Cell Carcinoma

In the context of OSCC, exosomes play significant roles that greatly influence both tumor progression and resistance to therapy. Exosomes can carry and transfer immunosuppressive molecules such as PD-L1 from tumor cells to immune cells, inducing T-cell exhaustion and reducing cytotoxic activity [34]. This mechanism facilitates immune evasion, allowing the tumor to thrive unchecked by the immune system, thereby supporting tumor persistence and progression [35].

Additionally, proteins and microRNAs (miRNAs) loaded in exosomes can reprogram cells within the TME, promoting angiogenesis, invasion, and cancer cell metastasis [36]. Oncogenic miRNAs transferred via exosomes, such as miR-21, is known to inhibit tumor suppressor genes in recipient cells, fostering malignant transformation and tumor progression [37]. This includes targeting pathways like PTEN and PDCD4 signaling, enhancing oncogenesis and metastatic behavior [38].

Exosomes can also transport contents that confer resistance to various treatment modalities, including chemotherapy and radiotherapy [35]. Indeed, they can carry RNAs or proteins that enhance DNA repair mechanisms or facilitate the efflux of chemotherapeutic drugs from cells, thereby reducing treatment efficacy [39]. For example, exosomes have been shown to transfer the MDR1/P-glycoprotein, a key player in drug resistance, which then promotes the expulsion of chemotherapeutic agents from cancer cells and diminishes their cytotoxic effects [40].

Furthermore, exosomes can alter the stromal environment and extracellular matrix to favor tumor invasion and metastasis [34,35]. They induce fibroblasts to convert into cancer-associated fibroblasts (CAFs), which secrete factors that further promote tumor progression. Signaling molecules and transforming growth factors carried by exosomes activate pathways such as TGF-β and SMAD in stromal cells, enhancing their supportive roles in tumor growth and survival [41].

Current studies emphasize the pivotal role exosomes play in OSCC pathology, highlighting their potential both as therapeutic targets and as biomarkers for disease monitoring. Consequently, deciphering the role of exosomes in the context of OSCC will open new avenues for therapeutic intervention, potentially leading to improved clinical outcomes for patients [42]. For instance, blocking the release of exosomes or modifying their content might be used as a strategy to suppress their ability to promote tumor growth and therapy resistance. Moreover, exosomes also have potential as relevant biomarkers for the diagnosis and prognosis of OSCC, reflecting the molecular composition and state of the originating tumor cells [42].

### 3.2. Oral Microbiome and Its Role in OSCC

The oral microbiome, encompassing the diverse community of microorganisms residing in the oral cavity, plays a significant role in maintaining oral health and has been increasingly recognized for its involvement in OSCC pathogenesis. Indeed, it is now well described that alterations in the composition and function of the oral microbiota, known as dysbiosis, can contribute to carcinogenesis through various mechanisms [43,44].

Chronic inflammation induced by pathogenic oral bacteria is a critical pathway linking the microbiome to OSCC development [45]. Persistent inflammatory responses lead to the production of pro-inflammatory cytokines, ROS, and reactive nitrogen species (RNS), which can cause DNA damage, promote genetic mutations, and create a pro-tumorigenic environment [46]. For instance, *Porphyromonas gingivalis*, a keystone pathogen in periodontal disease, has been implicated in OSCC development due to its ability to induce chronic inflammation and to modulate immune responses.

*Fusobacterium nucleatum* is another bacterium associated with OSCC, known for its pro-inflammatory properties and ability to adhere to and invade epithelial cells. In doing so, this microorganism can activate signaling pathways such as nuclear factor-kappa B (NF-κB) and extracellular signal-regulated kinase (ERK), leading to increased cell proliferation and inhibition of apoptosis. Moreover, *F. nucleatum* can modulate the immune microenvironment by attracting immunosuppressive cells and inhibiting cytotoxic immune responses, thereby facilitating tumor progression [47].

The oral microbiome also contributes to OSCC progression through the production of carcinogenic substances. Certain bacteria metabolize alcohol- and tobacco-derived compounds into acetaldehyde and other carcinogens, which can directly damage DNA and promote the malignant transformation of oral epithelial cells [48]. Additionally, microbial enzymes such as nitrosamines and lipid peroxidases can generate mutagenic compounds that further contribute to the carcinogenesis of the oral mucosal epithelium [49,50].

Furthermore, dysbiosis can disrupt the balance between beneficial and harmful microbial species, leading to decreased colonization resistance against pathogens and altered immune homeostasis [51,52]. This imbalance may enhance susceptibility to infections and inflammatory conditions that will predispose individuals to OSCC. Given those observations, recent studies have explored the potential of modulating the oral microbiome through probiotics, prebiotics, and antimicrobial therapies as both preventive and therapeutic strategies against OSCC [53,54].

## 4. Genetic and Molecular Determinants of OSCC

### 4.1. Genetic Alterations

#### 4.1.1. Oncogene TP53

**Figure 3:**
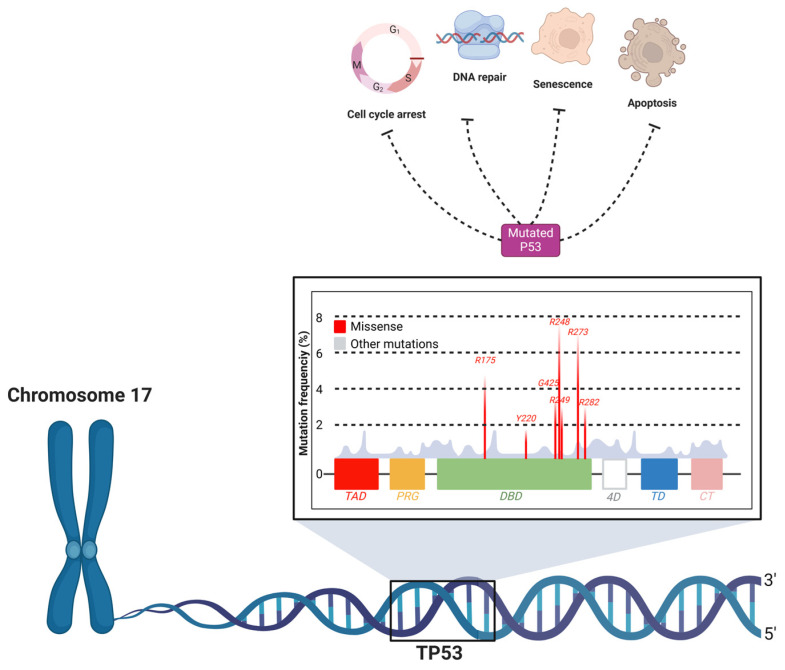
Mutation hotspots and functional impact of TP53 in oral cancer. The *TP53* gene, located on chromosome 17, encodes the p53 protein, a critical tumor suppressor that regulates cellular processes such as cell cycle arrest, DNA repair, senescence, and apoptosis. Mutations in *TP53* lead to dysfunctional p53 protein, which impairs these regulatory functions, contributing to carcinogenesis. The figure highlights the DNA-binding domain (DBD) as the primary region where missense mutations are concentrated, significantly impacting the tumor-suppressive capacity of p53. Common mutation hotspots (e.g., R175, R248, R273) are shown, which are frequently associated with OSCC. These mutations result in the loss of normal p53 activity, promoting the survival and proliferation of damaged cells.

The *TP53* gene is widely recognized as the “guardian of the genome” due to its crucial role in preserving genomic integrity [55,56]. The encoded p53 protein acts as a transcription factor that regulates a wide range of cellular processes, including cell cycle arrest, DNA repair, senescence, and apoptosis [57]. Under normal conditions, p53 responds to genotoxic stress, such as DNA damage, by activating mechanisms that prevent the proliferation of damaged cells [58]. However, mutations in the *TP53* gene result in a defective or inactive p53 protein (Figure 3), which is unable to perform its tumor suppressor functions [59].

Mutations in *TP53* are observed in approximately 50–70% of OSCC cases, making it one of the most prevalent genetic alterations associated with this type of cancer [8]. *TP53* gene mutations are predominantly missense mutations, and are mostly concentrated in the DNA-binding domain of the p53 protein, affecting its ability to regulate transcription and leading to the accumulation of mutant p53 protein due to impaired degradation [60]. Approximately 30% of these mutations occur at specific “hot spots” within the DNA-binding domain, which are critical for the gene’s tumor-suppressing function [60].

Other types of *TP53* mutations include nonsense mutations (which create a premature stop codon, leading to truncated non-functional protein), frameshift mutations (caused by insertions or deletions that alter the reading frame of the gene), and splice site mutations (which affect the splicing of pre-mRNA, potentially leading to the inclusion or exclusion of certain exons in the final mRNA product). These mutations generally result in a complete loss of function of the p53 protein, in contrast to the more common missense mutations, which often retain some residual activity [58]. As a result, there is a loss of the ability to induce cell cycle arrest and apoptosis, allowing cells with genomic damage to proliferate uncontrollably [59]. Furthermore, research by Stransky et al. (2011) highlighted that those mutations in *TP53* are strongly associated with genomic instability, a phenomenon that facilitates the acquisition of other oncogenic mutations and contributes to tumor heterogeneity [61].

The p53 protein interacts with a variety of proteins to perform its critical role in maintaining cellular integrity and preventing cancer development. One of the most significant interactions is with MDM2 (Mouse Double Minute 2), a protein that negatively regulates p53 by promoting its degradation through ubiquitination [62]. This interaction is crucial for keeping p53 levels low under normal conditions and allowing for rapid activation in response to cellular stress, such as DNA damage [62].

Another important interaction is with p21 (Cyclin-Dependent Kinase Inhibitor 1A), which is induced by p53. p21 inhibits cyclin-dependent kinases (CDKs), leading to cell cycle arrest, particularly at the G1/S checkpoint. This pause in the cell cycle allows the cell time to repair DNA damage or, if the damage is too extensive, to proceed toward apoptosis, thereby preventing the proliferation of potentially cancerous cells [63].

p53 also interacts with the pro-apoptotic proteins BAX (Bcl-2-associated X protein) and PUMA (p53 Upregulated Modulator of Apoptosis) [64]. BAX and PUMA promote apoptosis by facilitating the release of cytochrome *c* from mitochondria, which triggers the activation of caspases via the apoptosome pathway, leading to cell death. This apoptotic pathway is particularly important for eliminating cells that have accumulated significant genetic damage [64].

Finally, p53 regulates the expression of GADD45 (Growth Arrest and DNA-Damage-inducible 45), a protein involved in DNA repair and cell cycle arrest [62]. GADD45 helps maintain genomic stability by allowing time for the repair of damaged DNA, further preventing the accumulation of mutations that could lead to cancer [62].

Genomic instability, caused by p53 dysfunction, plays a central role in OSCC carcinogenesis [65]. It not only promotes the accumulation of additional genetic mutations, but also allows clonal evolution within the tumor, resulting in cellular subpopulations with distinct characteristics, such as resistance to specific treatments and increased metastatic potential [65]. Loss of p53 function has also been associated with chronic inflammation and modulation of the TME, creating conditions that favor tumor progression and invasion [66].

##### Innovative Therapeutic Strategies for p53 Restoration

Innovative therapeutic strategies are currently being explored to restore the function of p53 [67]. Among these strategies, the use of small molecules and gene therapy stand out, both being aimed at reactivating the tumor suppressor activity of p53 [67,68].

APR-246, also known as eprenetapopt, is one of the most promising agents in this field. APR-246 was developed to convert mutant p53 into its active conformation, recovering its ability to transcribe genes responsible for apoptosis and cell cycle arrest [69,70]. This reactivation allows p53 to effectively respond to genotoxic stress, leading damaged cells to apoptosis instead of allowing their proliferation [69]. Clinical studies have demonstrated that APR-246, especially when used in combination with chemotherapy, significantly improves tumor responses in patients with OSCC, reflected not only in the suppression of tumor growth, but also in extended survival rates [71].

In addition to small molecules, gene therapy presents a robust approach, directly inserting a functional copy of the TP53 gene into tumor cells via viral vectors [72,73]. This strategy aims to replace defective p53 with a functional version that can re-establish cell cycle checkpoint mechanisms and induce programmed cell death in tumor cells [72]. Recent research has shown that this approach can lead to a significant reduction in tumor volume and improvement in survival rates in pre-clinical models, highlighting its therapeutic potential [74,75].

The implementation of these therapies in clinical settings requires a detailed understanding of their mechanisms and a rigorous assessment of their efficacy and safety [74]. The identification of biomarkers that can accurately predict the therapeutic response to these treatments could also allow for a more OSCC-patient-personalized approach, maximizing therapeutic efficacy while minimizing adverse effects, hence improving the outcomes of this aggressive form of cancer [75,76].

#### 4.1.2. Oncogene RAS

The *RAS* genes, which include *H-RAS*, *K-RAS*, and *N-RAS*, encode a family of GTPase proteins that are crucial in the transduction of growth and survival cellular signals [77]. The RAS proteins act as molecular switches, alternating between active (GTP-bound) and inactive (GDP-bound) states [78]. In their active form, RAS proteins interact with various downstream signaling pathways, such as the MAPK/ERK and the PI3K/AKT pathways, promoting cell proliferation and survival [79,80].

**Figure 4:**
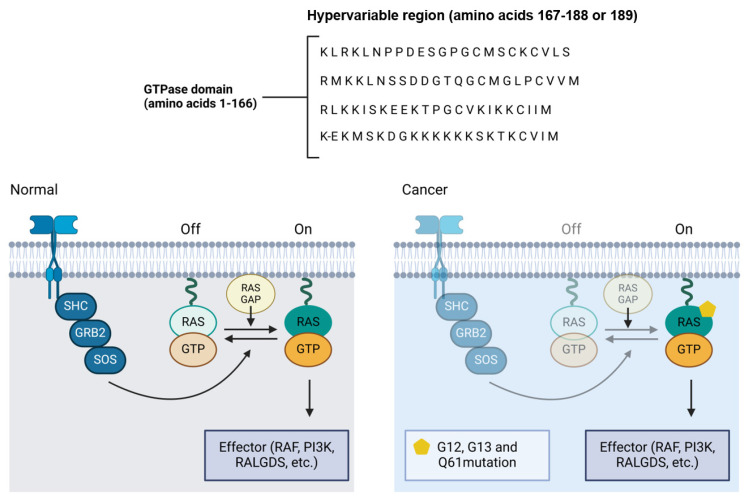
RAS pathway mutation and its implications in oral cancer. The normal and mutated states of the RAS signaling pathway, illustrating how mutations at codons 12, 13, or 61 in the RAS gene result in constitutively active RAS proteins. These mutations prevent RAS from switching off, leading to the continuous activation of downstream effectors such as RAF, PI3K, and RALGDS, promoting unchecked cell proliferation. In OSCC, although RAS mutations are less frequent compared to other cancers, their presence can contribute to resistance to tyrosine kinase inhibitor therapies, complicating treatment strategies.

Mutations in the *RAS* genes, though less frequent in OSCC compared to other types of cancers, play a significant role when present (Figure 4). *RAS* mutations often occur at codons 12, 13, or 61, resulting in the expression of constitutively active RAS proteins that continuously signal for cell proliferation, regardless of external stimuli [81]. Research by Cohen et al. (2010) suggests that the aberrant activation of the RAS pathway may also contribute to resistance to tyrosine kinase inhibitor based therapies, which are commonly used in the treatment of OSCC [82].

The continuous activation of RAS results in the persistent stimulation of growth signaling pathways, such as the MAPK/ERK pathway, which promotes cell proliferation and survival, and the pro-survival PI3K/AKT pathway, which inhibits apoptosis [80]. These pathways not only facilitate the uncontrolled proliferation of cancer cells, but also confer an adaptive advantage to the tumor, allowing it to adapt and survive under adverse conditions, such as the presence of chemotherapeutic agents. Additionally, RAS activation is associated with increased angiogenesis, invasion, and metastasis potential, allowing tumor progression and cancer dissemination [83].

Recent advances in targeted cancer therapies have led to the development of small molecule inhibitors designed to address oncogenic *RAS* gene mutations, particularly regarding *KRAS*, which is the most frequently mutated *RAS* isoforms in human cancers [76,84]. One of the most notable breakthroughs has been the development of covalent inhibitors targeting the *KRAS* G12C mutation, a substitution that results in a constitutively active KRAS protein, driving oncogenic signaling [85,86]. Among these inhibitors, sotorasib and adagrasib have emerged as first-in-class agents capable of selectively and irreversibly binding to the cysteine residue introduced by the G12C mutation, locking KRAS in its inactive GDP-bound state and thereby inhibiting downstream signaling through the MAPK/ERK and PI3K/AKT pathways [86]. Clinical trials have demonstrated the significant efficacy of these inhibitors in non-small-cell lung cancer (NSCLC) harboring the KRAS G12C mutation, with improved progression-free survival and satisfactory safety profiles. For instance, the phase II CodeBreaK 100 trial with sotorasib showed a disease control rate of approximately 80% in pretreated NSCLC patients [86].

Unfortunately, the clinical utility of KRAS G12C inhibitors in oral OSCC remains limited due to the very low frequency of KRAS G12C mutations in this tumor type [81]. Comprehensive genomic analyses of OSCC have indeed shown that RAS mutations are relatively rare events, and, when present, they more commonly involve H-RAS, rather than KRAS, and often affect different codons (e.g., 12, 13, or 61) [87]. Consequently, the current generation of KRAS G12C-specific inhibitors has limited applicability for most OSCC patients [85].

These observations underscore the importance of not only developing inhibitors that can target a broader spectrum of RAS mutations—including H-RAS and K-RAS variants beyond G12C—but also identifying alternative therapeutic strategies [85,87]. Such strategies may include targeting downstream effectors of RAS signaling, such as MEK, ERK, or PI3K, or modulating upstream receptor pathways (e.g., EGFR), which can indirectly influence RAS activity [87]. Moreover, combination therapies that integrate RAS-pathway inhibitors with immunotherapy or chemotherapy are being investigated to overcome resistance mechanisms and enhance treatment efficacy [88].

Thus, while KRAS G12C inhibitors represent a significant milestone in precision oncology, their impact on OSCC is currently limited, emphasizing the need for more precise molecular profiling to uncover new therapeutic vulnerabilities within the RAS signaling axis in this tumor type [85,86,88].

#### 4.1.3. Oncogenic Signaling in Oral Squamous Cell Carcinoma

OSCC is characterized by the dysregulation of several key signaling pathways that contribute to tumor initiation, progression, and resistance to therapy [14,61,83,89]. Among the most critical pathways implicated in OSCC are the EGFR, PI3K/AKT/mTOR, JAK/STAT, and Wnt/β-catenin signaling pathways. Each of these pathways plays a unique role in the oncogenesis of OSCC [1,12,83,90,91].

The epidermal growth factor receptor (EGFR) pathway is frequently activated in OSCC and is associated with aggressive tumor behavior and poor prognosis [63]. EGFR, a transmembrane tyrosine kinase receptor, is overexpressed or mutated in many OSCC cases, leading to the continuous activation of downstream signaling cascades that promote cell proliferation, survival, angiogenesis, and metastasis [92]. The aberrant activation of EGFR primarily triggers the MAPK, PI3K/AKT, and JAK/STAT pathways, which collectively enhance tumor cell proliferation and inhibit apoptosis [93]. Targeted therapies against EGFR, such as cetuximab, have been explored in OSCC treatment, though resistance often develops due to alternative pathway activation or mutations in downstream effectors [94].

The PI3K/AKT/mTOR pathway is a central regulator of cell growth, survival, and metabolism, and is frequently dysregulated in OSCC [95]. The activation of this pathway often results from mutations or amplifications in *PIK3CA* (the gene encoding the catalytic subunit of PI3K) or the loss of function of PTEN, a tumor suppressor that negatively regulates PI3K signaling [96,97]. The resulting hyperactivation of PI3K leads to the phosphorylation and activation of AKT, which subsequently activates mTOR, promoting protein synthesis, cell growth, and survival [98,99]. Aberrations in this pathway, therefore, contribute to the resistance of OSCC to conventional therapies, making it a target for new therapeutic strategies, including mTOR inhibitors and PI3K inhibitors [100].

The Janus kinase (JAK)/signal transducer and activator of transcription (STAT) pathway is another key signaling mechanism implicated in OSCC aggressiveness [101]. The activation of this pathway often occurs via cytokines and growth factors binding to their specific receptors, leading to the phosphorylation and activation of JAKs, which, in turn, phosphorylate STAT proteins [102,103]. Phosphorylated STATs, then, dimerize and translocate to the nucleus, where they regulate the expression of genes involved in cell proliferation, survival, and immune evasion [104]. In OSCC, the constitutive activation of the JAK/STAT pathway, particularly STAT3, has been associated with increased tumor growth, resistance to apoptosis, and immune suppression within the TME [105]. Considering those observations, inhibitors targeting JAKs or STAT3 are currently being clinically explored as potential therapeutic options for OSCC treatment [101,102].

The Wnt/β-catenin signaling pathway plays a crucial role in cellular differentiation, proliferation, and migration [106]. In the context of OSCC, the aberrant activation of the Wnt/β-catenin pathway has been linked to enhanced tumor growth, invasion, and resistance to therapy [107]. This pathway is activated when Wnt ligands bind to Frizzled receptors, preventing the degradation of β-catenin. Accumulated β-catenin translocates to the nucleus and activates the transcription of target genes involved in cell cycle progression and survival [108]. Mutations in components of the Wnt pathway or dysregulation of β-catenin degradation mechanisms can lead to sustained pathway activation in OSCC. Given its crucial role in stem cell maintenance and the tumorigenesis of OSCC, the Wnt/β-catenin pathway represents a promising target for therapeutic intervention in this oral malignancy [109].

### 4.2. Epigenetic

#### 4.2.1. DNA Methylation

Among the epigenetic mechanisms, DNA methylation is one of the most studied and plays a significant role in the carcinogenesis of OSCC [110,111]. DNA methylation primarily occurs at cytosines located in CpG dinucleotides, where the addition of methyl groups alters chromatin structure and, consequently, regulates gene expression [112].

DNA methylation is mediated by enzymes known as DNA methyltransferases (DNMTs), which transfer methyl groups from S-adenosylmethionine (SAM) to the 5-position of the cytosine ring [113]. This process can result in the transcriptional repression of specific genes, particularly those with CpG-rich islands in their promoter regions. When these CpG islands are hypermethylated, gene expression is often silenced, which can affect tumor suppressor genes and other critical pathways in carcinogenesis [112].

Several tumor suppressor genes are frequently silenced in OSCC and other cancers due to CpG island hypermethylation [114,115]. One such gene is *RASSF1A*, encoding the well-known tumor suppressor RASSF1 (Ras Association Domain Family Member 1), which plays a pivotal role in the regulation of cell cycle arrest and apoptosis [116]. The hypermethylation of the *RASSF1A* promoter is commonly observed in OSCC and leads to its transcriptional silencing, contributing to unchecked cellular proliferation and tumor progression [116].

Another key gene frequently silenced by hypermethylation is *APC* (Adenomatous polyposis coli), which is involved in the Wnt signaling pathway [117]. Loss of *APC* function due to promoter hypermethylation disrupts the regulation of β-catenin, leading to the aberrant activation of the Wnt pathway and promoting cell proliferation and invasion in cancerous tissues [117]. Similarly, *MLH1* (MutL Homolog 1), a gene critical for DNA mismatch repair, is often hypermethylated in OSCC, leading to a deficiency in DNA repair mechanisms [118]. This results in increased mutation rates, genomic instability, and a higher likelihood of cancer progression [119].

Aberrant DNA methylation is a common feature in many types of cancer, including OSCC [120,121,122], and numerous studies have shown that the hypermethylation of tumor suppressor gene promoters is an early event in OSCC carcinogenesis [122]. For example, the hypermethylation of the *p16INK4a* (also known as *CDKN2A*; Cyclin-dependent kinase inhibitor 2A) gene promoter, a crucial cell cycle regulator, leads to its silencing and results in uncontrolled cell proliferation [123]. Leemans et al. (2011) reported that *p16INK4a* promoter hypermethylation is frequently observed in OSCC, contributing to cell cycle deregulation and tumor progression [8].

Notably, DNA methylation is involved in the early stages of OSCC [124]. In tobacco users, exposure to carcinogens such as polycyclic aromatic hydrocarbons and nitrosamines induces aberrant methylation patterns in critical genes that regulate cell cycle control, apoptosis, and DNA repair [125]. The hypermethylation of tumor suppressor genes like *p16INK4a* and *RASSF1A* is frequently observed in pre-malignant tissues of tobacco users. Indeed, a number of studies have shown that approximately 40–60% of pre-malignant oral tissues in smokers exhibit the aberrant methylation of *p16INK4a*, leading to the silencing of this key gene and contributing to uncontrolled cell proliferation and tumorigenesis [126]. These methylation changes provide an early indicator of genomic instability and play a pivotal role in the progression from dysplasia to invasive carcinoma in the context of tobacco-induced carcinogenesis [127].

In addition to *p16INK4a*, the aberrant methylation of other tumor suppressor genes, such as *MGMT* (O6-methylguanine-DNA methyltransferase), *RASSF1A*, and *DAPK* (death-associated protein kinase), has also been documented in OSCC [128,129,130]. Stransky et al. (2011) identified that *MGMT* promoter hypermethylation is associated with reduced DNA repair capacity, increasing susceptibility to additional mutations and contributing to genomic instability [91].

**Figure 5:**
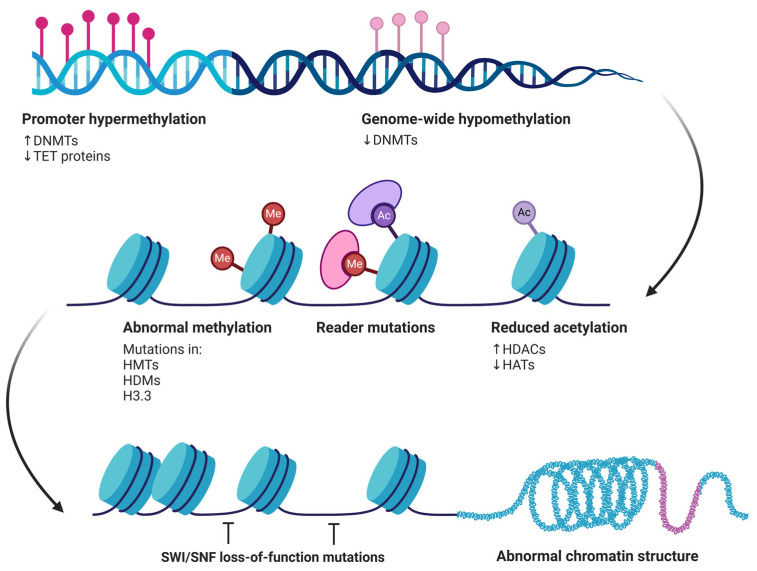
Epigenetic alterations contributing to cancer development. Promoter hypermethylation, driven by increased DNMT activity and reduced TET protein function, silences tumor suppressor genes. Concurrently, genome-wide hypomethylation results from decreased DNMTs, leading to genomic instability. Other alterations include abnormal methylation patterns, caused by mutations in histone methyltransferases (HMTs), histone demethylases (HDMs), and histone variant H3.3. Reader mutations affect the proteins that interpret these methylation marks, while reduced acetylation, due to increased histone deacetylases (HDACs) and decreased histone acetyltransferases (HATs), further disrupts gene regulation. Together, these modifications contribute to abnormal chromatin structure and loss of SWI/SNF function, ultimately promoting oncogenesis.

The functional consequences of aberrant DNA methylation include the inactivation of genes critical for tumor suppression (Figure 5), cell cycle control, apoptosis, DNA repair, and other essential cellular functions [122,131]. Silencing tumor suppressor genes through DNA hypermethylation allows cancer cells to escape normal control mechanisms, promoting proliferation, survival, and invasion [121]. Additionally, aberrant methylation can interact with other genetic and epigenetic alterations to drive cancer progression [121]. The elegant study by Chen et al. (2021) highlighted the importance of epigenetic modifications, including DNA methylation, in regulating gene expression in head and neck cancers [12]. Interestingly, they observed that reversing aberrant methylation through demethylating agents can restore the expression of tumor suppressor genes, offering a potential strategy for epigenetic therapies [131].

#### 4.2.2. Histone Modifications

Histone modifications are crucial epigenetic regulations influencing chromatin structure and, consequently, gene expression [132,133]. In the context of OSCC, these modifications play a significant role in carcinogenesis, affecting various cellular processes essential for maintaining cellular homeostasis and preventing malignant transformation [134,135].

Histones are proteins that help pack DNA in the cell nucleus, playing critical roles in chromatin assembly and compaction by forming the nucleosome [132]. The main histone modifications include acetylation, methylation, phosphorylation, ubiquitination, and sumoylation [132]. These modifications occur at specific histone residues, mainly on the N-terminal tails, and are mediated by specific enzymes such as histone acetyltransferases (HATs), histone deacetylases (HDACs), histone methyltransferases (HMTs), and histone demethylases (HDMs) [134].

##### Histone Acetylation

Histone acetylation is one of the most studied modifications in cancer [132]. It is mediated by HATs, which add acetyl groups to lysines on histone tails, generally resulting in a more relaxed and transcriptionally active chromatin [136]. In contrast, HDACs remove these acetyl groups, leading to more condensed chromatin and gene repression [137]. In OSCC, the dysregulation of the balance between histone acetylation and deacetylation has been largely implicated in carcinogenesis [135], and recent studies have shown that the aberrant expression of HDACs was associated with OSCC progression. The overexpression of HDACs indeed can lead to the repression of tumor suppressor genes, promoting cell proliferation and resistance to apoptosis [134].

Interestingly, HDAC inhibitors (HDACis) have shown therapeutic potential by restoring the expression of tumor suppressor genes and inducing apoptosis in OSCC cells [134]. Consequently, HDACis have emerged as promising therapeutic agents in the treatment of various cancer settings, including OSCC [138,139]. By inhibiting HDAC activity, HDACis promote a more relaxed chromatin structure, thereby reactivating the expression of tumor suppressor genes and inducing apoptosis in cancer cells [138,139]. Several HDACis have been approved by the United States Food and Drug Administration (FDA) for cancer therapy [140,141,142]. For instance, vorinostat (suberoylanilide hydroxamic acid, SAHA) was the first HDACi approved for the treatment of cutaneous T-cell lymphoma (CTCL) [141]. Similarly, belinostat (PXD101) [142] and panobinostat (LBH-589) [140] have received FDA approval for the treatment of peripheral T-cell lymphomas (PTCL) and multiple myeloma, respectively. In the context of OSCC, both preclinical and clinical studies have investigated the efficacy of HDACis [139]. Preclinical evidence has shown that HDACis can induce apoptosis, cause cell cycle arrest, and reduce the proliferation and metastatic potential of OSCC cells [143]. One study reported that the combination of low concentrations of DNA methyltransferase inhibitors (DNMTis), histone methyltransferase inhibitors (HMTis), and HDACis effectively reduced OSCC cell viability by inducing apoptosis and causing cell cycle arrest at the S and G2/M phases [144].

##### Histone Methylation

Histone methylation can either activate or repress gene transcription, depending on the lysine or arginine residue that is methylated, and the number of methyl groups added (mono-, di-, or trimethylation) [145,146]. In OSCC, dysregulated histone methylation has been associated with changes in the expression of critical genes involved in carcinogenesis [147]. The trimethylation of lysine 27 on histone H3 (H3K27me3), mediated by the enzyme EZH2 (Enhancer of zeste homolog 2), is a marker of gene repression [148,149]. The overexpression of EZH2 and increased H3K27me3 have been correlated with tumor progression and poor prognosis in OSCC [149].

Zheng et al. (2019) have reported that EZH2 overexpression is associated with the repression of tumor suppressor genes, contributing to cell proliferation and resistance to apoptosis in OSCC cells [150]. Furthermore, the inhibition of EZH2 results in the reactivation of tumor suppressor genes and the inhibition of tumor growth, suggesting that EZH2 is a promising therapeutic target [150].

Histone modifications not only regulate gene expression, but also offer therapeutic opportunities [150,151]. Indeed, the use of HDAC inhibitors and histone methyltransferase inhibitors (HMTis) has shown promising results in preclinical models of OSCC [152]. These therapeutic agents aim to restore the expression of tumor suppressor genes and reverse the malignant phenotype of cancer cells [152,153]. Clinical studies are currently ongoing to evaluate the efficacy of these inhibitors in combination with other therapies, such as chemotherapy and immunotherapy [152].

#### 4.2.3. Regulation by microRNAs

MicroRNAs (miRNAs) are small non-coding RNA molecules, about 18–25 nucleotides in length, that play crucial roles in the post-transcriptional regulation of gene expression [154,155,156]. In the context of OSCC, miRNAs have emerged as important regulators of various biological and pathological processes, including cell proliferation, apoptosis, angiogenesis, and metastasis [157,158,159].

miRNAs regulate gene expression by binding to complementary sequences on target messenger RNAs (mRNAs), usually in the 3′ untranslated region (3′ UTR) [155]. This binding results in mRNA degradation or translation inhibition, depending on the degree of complementarity [154]. Therefore, the dysregulation of miRNAs can alter the expression of critical genes involved in carcinogenesis, contributing to the malignant transformation of cells [156].

**Figure 6:**
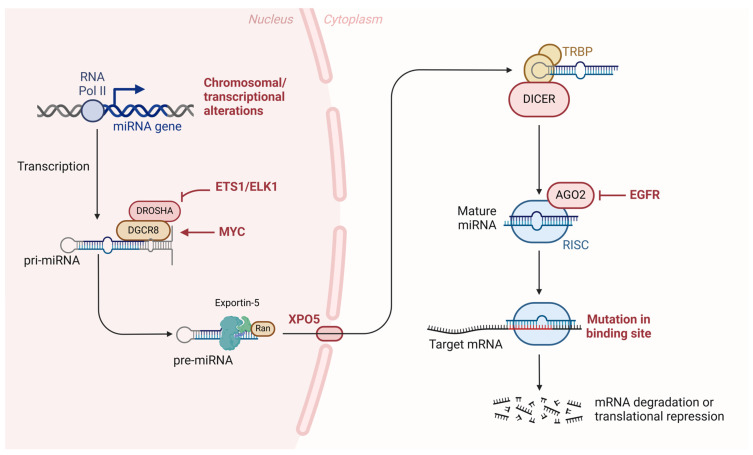
Role of microRNAs in oral cancer progression. The transcription of miRNA genes by RNA polymerase II leads to the formation of primary miRNAs (pri-miRNAs), which undergo processing by the DROSHA/DGCR8 complex into precursor miRNAs (pre-miRNAs). Exportin-5 (XPO5) transports pre-miRNAs to the cytoplasm, where DICER and TRBP further process them into mature miRNAs. The mature miRNAs associate with the RNA-induced silencing complex (RISC), guiding the complex to target mRNA for degradation or translational repression. Mutations affecting key components of this pathway, including the binding sites of target mRNAs or alterations in regulatory elements such as EGFR and transcription factors like MYC and ETS1/ELK1, contribute to the dysregulation of gene expression and promote oral cancer development.

Several studies have identified specific miRNAs that are dysregulated in OSCC (Figure 6), with important roles in cancer pathogenesis and progression [160,161,162,163]. miR-21 is one of the most frequently overexpressed miRNAs in various types of cancer, including OSCC. It acts as an oncomir, promoting cell survival and resistance to apoptosis by targeting tumor suppressor genes such as *PTEN* and *PDCD4* (programmed cell death protein 4) [164]. Importantly, miR-21 overexpression is associated with poor prognosis in patients with OSCC [164].

Recently, it has been demonstrated that miR-21 promotes the activation of tumor-associated macrophages (TAMs) in OSCC, resulting in a pro-tumor phenotype [165]. This process favors immune evasion and contributes to tumor progression. Modulating miR-21 expression in macrophages can directly impact the efficacy of immunotherapies, opening new therapeutic possibilities [165].

Another relevant aspect is the role of miR-21 in regulating oxidative stress response. Studies such as Cheng et al.’s (2021) demonstrated that, in OSCC cells, miR-21 can induce greater resistance to oxidative stress, which is an important adaptive characteristic that allows tumor cells to survive in hostile environments, such as after radiotherapy [166]. This observation makes miR-21 an interesting target for therapies aimed at increasing tumor sensitivity to oxidative stress and improving treatment response.

The miRNA miR-34a plays a crucial role as a tumor suppressor in various cancers, including OSCC. Its activity is directly linked to the *TP53* gene as miR-34a is one of its key transcriptional targets [167]. Once activated, miR-34a can induce cell cycle arrest, apoptosis, and the suppression of cell proliferation, acting as a consistent tumor suppressor [168].

Recent studies, such as Li et al. (2021), have shown that reduced miR-34a expression correlates with a poor prognosis in OSCC patients [169]. This is partly due to the epigenetic silencing of miR-34a, primarily through promoter hypermethylation, which is a common feature in head and neck tumors [169]. The subsequent loss of miR-34a expression promotes uncontrolled cell proliferation and facilitates tumor progression by impairing the cells’ ability to activate apoptosis [169]. In addition, miR-34a negatively regulates several proteins involved in tumor invasion and metastasis. Specifically, miR-34a inhibits the expression of epithelial–mesenchymal transition (EMT)-related genes such as *SNAIL* (Snail Family Transcriptional Repressor) and *ZEB1* (Zinc finger E-box-binding homeobox 1), which are critical for the metastatic spread of tumor cells. The resulting miR-34a-reduced expression renders these pro-invasive pathways more active [40].

The miRNA miR-155 expression is frequently elevated in OSCC, where it promotes cell proliferation and invasion by negatively regulating targets such as *SOCS1* (Suppressor of Cytokine Signaling 1) and C/*EBPβ* (CCAAT Enhancer Binding Protein Beta) [170]. miR-155 overexpression is associated with tumor aggressiveness and poor prognosis [171]. SOCS1 is a crucial negative regulator of the JAK/STAT signaling pathway, which is involved in mediating immune responses, cell growth, and survival [172]. By targeting *SOCS1*, miR-155 leads to the uncontrolled activation of the JAK/STAT pathway, promoting cell proliferation and survival in OSCC [173]. A loss of SOCS1 also contributes to immune evasion by cancer cells, as this pathway is involved in modulating the immune response against tumor cells. Indeed, miR-155-mediated downregulation of SOCS1 has been associated with increased tumor aggressiveness and resistance to immune checkpoint inhibitors in OSCC [174]. Another important target of miR-155 is CCAAT/enhancer-binding protein beta (C/EBPβ), a transcription factor involved in cell differentiation and immune regulation. C/EBPβ plays a role in promoting cell cycle arrest and differentiation, acting as a tumor suppressor [175]. By downregulating C/EBPβ, miR-155 promotes tumor progression through enhanced proliferation and inhibition of differentiation, contributing to a more aggressive phenotype in OSCC [176]. The suppression of C/EBPβ also impairs the body’s immune response to the tumor, further facilitating the tumor’s escape from immune surveillance [177].

The miRNA miR-200c is involved in the regulation of epithelial–mesenchymal transition (EMT), a critical process in cancer metastasis [178,179]. Research by Song et al. (2020) has revealed that reduced miR-200c expression in OSCC is associated with increased invasion and metastasis through the dysregulation of targets such as ZEB1 and ZEB2 [180]. The EMT is a crucial process in the metastasis of OSCC [181,182]. During EMT, epithelial cells, which normally exhibit strong cell–cell adhesion and a structured arrangement, lose these characteristics and acquire mesenchymal traits, including enhanced mobility, invasiveness, and resistance to apoptosis [183,184]. This transition allows cancer cells to detach from the primary tumor, invade surrounding tissues, and enter the bloodstream or lymphatic system, which is essential for the metastatic spread of OSCC to distant organs [185].

In OSCC, the activation of EMT is driven by key transcription factors such as SNAIL, SLUG (also called SNAI2), Snail family transcriptional repressor 2, TWIST, and ZEB1/2, which repress epithelial markers like E-cadherin, a protein essential for maintaining cell adhesion [183]. The downregulation of E-cadherin and other epithelial markers enables tumor cells to gain mesenchymal properties, facilitating invasion and dissemination [185]. Importantly, EMT not only supports the initial escape of tumor cells from the primary site, but also contributes to their survival in circulation and colonization at distant metastatic sites, making it a critical step in OSCC metastasis [184].

### 4.3. Genomic Instability

#### 4.3.1. Mutations

Genomic instability is a fundamental characteristic of cancer and plays a central role in the carcinogenesis of OSCC [90,186]. It refers to the high frequency of genetic alterations in a cell genome, including point mutations, chromosomal rearrangements, deletions, insertions, and aneuploidy [186]. These genetic events contribute to tumor heterogeneity and the ability of cancer cells to adapt to various environmental and therapeutic stresses [90].

#### 4.3.2. Types of Mutations

The mutations occurring in OSCC can be classified into several types, each with different impacts on cellular function and tumor progression.

##### Point Mutations

Point mutations are alterations in the DNA sequence that affect a single nucleotide. These mutations can be missense (substitution of one amino acid for another), nonsense (introduction of a premature stop codon), or silent (no change in the amino acid sequence) [187]. In OSCC, point mutations in oncogenes and tumor suppressor genes are common. As mentioned above, mutations in the *TP53* gene are frequently observed, resulting in a dysfunctional p53 protein that cannot regulate apoptosis and DNA repair [188]. Point mutations in TP53 are present in approximately 60% of OSCC cases and are associated with the loss of p53 tumor suppressor function, contributing to uncontrolled cell proliferation and resistance to apoptosis [189].

##### Chromosomal Rearrangements

Chromosomal rearrangements, including translocations, inversions, and duplications, are pivotal genetic events in the pathogenesis of OSCC [190]. These rearrangements can lead to the aberrant activation of oncogenes or the inactivation of tumor suppressor genes, thereby disrupting normal cellular regulatory mechanisms. Such genetic alterations may result in the formation of fusion genes or modifications in the transcriptional regulation of critical genes involved in cell cycle control, apoptosis, and DNA repair. These disruptions contribute to tumorigenesis, disease progression, and therapeutic resistance in OSCC [61]. For example, rearrangements involving the *CCND1* gene, which encodes cyclin D1, a key regulator of the G1-S phase transition in the cell cycle, can result in its overexpression, promoting cell cycle progression and tumor proliferation [16,20,191]. Amplifications or translocations at the CCND1 locus result in its overexpression, leading to dysregulated cell cycle progression, increased cellular proliferation, and enhanced tumor aggressiveness. Importantly, the overexpression of cyclin D1 has been linked to poor prognosis and resistance to standard therapies in OSCC, as highlighted by Leemans et al. (2018) [192]. In addition to *CCND1*, rearrangements involving genes such as *PIK3CA* and *EGFR* are also implicated in OSCC pathophysiology, contributing to tumor progression and reduced therapeutic efficacy, thereby representing potential targets for novel treatment strategies [193].

##### Deletions and Insertions

Deletions and insertions of DNA segments can result in the loss or gain of function of crucial proteins [59]. The deletion of tumor suppressor genes, such as *CDKN2A*, which encodes the p16INK4a protein, is common in OSCC and is associated with cell cycle dysregulation and uncontrolled cell proliferation [194]. Homozygous deletions of *CDKN2A* were found in a significant percentage of OSCC cases, resulting in the loss of cell cycle regulation and contributing to the malignant transformation of cells [195].

Another important gene frequently altered in OSCC is *NOTCH1* (Notch Receptor 1), which plays a key role in cell differentiation and the maintenance of epithelial tissues. *NOTCH1* mutations and deletions are associated with epithelial dysplasia and the initiation and progression of OSCC [91,196]. Various studies have shown that deleterious mutations in *NOTCH1* occur in early stages of OSCC and are linked to poor differentiation and aggressive tumor behavior [196].

Furthermore, *PTEN*, a tumor suppressor gene that encodes a lipid phosphatase, is often deleted or mutated in OSCC. The loss of *PTEN* leads to the activation of the PI3K/AKT signaling pathway, which promotes cell survival and proliferation. Alterations in PTEN are associated with poor prognosis in OSCC patients [99,100].

Finally, *CCND1* (Cyclin D1) is another gene frequently affected by amplifications and rearrangements. While amplifications of *CCND1* are well known to drive cell cycle progression, insertions in regulatory regions can also lead to its overexpression, contributing to uncontrolled cell proliferation in OSCC [197].

##### Aneuploidy

Aneuploidy is defined by the presence of an abnormal number of chromosomes in a cell [198]. In OSCC, aneuploidy is frequently observed and is associated with genomic instability, promoting genetic variability within the tumor and contributing to the adaptation and evolution of cancer cells [198]. Research by Tang et al. (2022) showed that aneuploidy in OSCC correlates with tumor aggressiveness and poorer prognosis [199]. Aneuploidy can result from the failure of proper chromosome segregation during mitosis, leading to cells with gains or losses of entire chromosomes, which can affect the expression of genes involved in cell proliferation and survival [200].

Additionally, aneuploidy fosters tumor heterogeneity, creating a diverse population of cancer cells within the TME [201]. This diversity allows some cells to survive environmental challenges and adapt to changes in the TME, such as hypoxia, acidity, and altered metabolic demands [202]. Cancer cells exhibiting chromosomal gains may overexpress genes involved in proliferation and survival, such as cyclins and growth factor receptors, while cells with chromosomal losses might eliminate tumor suppressor genes, further enhancing malignancy. This dynamic genomic evolution driven by aneuploidy plays a critical role in enabling OSCC cells to invade adjacent tissues, metastasize, and develop resistance to treatment [203]. The TME also actively shapes the evolution of cancer cells. Factors such as inflammatory cytokines, hypoxia, and nutrient deprivation impose selective pressures that influence the survival of certain aneuploid subclones [204]. As these subclones adapt, they may further remodel the TME to become more favorable for cancer progression, for example, by recruiting tumor-associated macrophages (TAMs) or promoting angiogenesis to sustain nutrient supply [205]. This reciprocal relationship between aneuploid cancer cells and their surrounding microenvironment accelerates the evolution of highly aggressive phenotypes, leading to tumor progression and poor clinical outcomes in OSCC [206].

##### Hypoxia

Hypoxia plays a pivotal role in the TME of OSCC by stabilizing and activating hypoxia-inducible factor 1-alpha (HIF-1α) [21,207,208]. This transcription factor crucially influences several downstream genes that are integral to the tumorigenic processes, such as angiogenesis, metabolism, cell survival, and invasion, each being essential for tumor progression [208]. Under hypoxic conditions, HIF-1α enhances the expression of vascular endothelial growth factor (VEGF), which is a primary mediator of angiogenesis [209]. This upregulation helps in fostering tumor growth by improving the supply of nutrients and oxygen to the tumor [208]. Moreover, hypoxia triggers a metabolic shift from oxidative phosphorylation to glycolysis, a phenomenon known as the Warburg effect, also orchestrated by the activation of HIF-1α [210]. This metabolic adaptation enables cancer cells to survive even under low oxygen levels. Additionally, hypoxia facilitates the epithelial–mesenchymal transition (EMT), thereby increasing the invasiveness of cancer cells [211]. In this process, HIF-1α is instrumental in upregulating EMT markers while concurrently downregulating epithelial markers, promoting metastasis [212,213].

Finally, the hypoxic conditions prevalent in OSCC also contribute to therapeutic resistance, particularly to radiation and chemotherapy [214]. This resistance primarily emerges because hypoxia diminishes the efficacy of treatments that rely on oxygen to generate ROS, thereby challenging the current therapeutic approaches and highlighting the need for novel strategies that can effectively target and mitigate the hypoxic microenvironment in OSCC [215,216].

#### 4.3.3. Consequences of Mutations

The mutations contributing to genomic instability in OSCC have several functional consequences that facilitate malignant transformation and cancer progression.

Mutations in genes regulating the cell cycle, such as TP53 and CDKN2A, result in the loss of control over cell proliferation, allowing cancer cells to divide uncontrollably. For example, the loss of p16INK4a function leads to the unchecked activation of cyclin-CDKs, promoting deregulated cell cycle transition from the G1 to the S phase 167.

Alterations in genes involved in apoptosis, such as TP53 and BAX, allow cancer cells to avoid programmed cell death, contributing to the survival and accumulation of mutated cell. Mutations in TP53 have been associated with reduced expression of the proapoptotic factor BAX, resulting in apoptosis evasion and resistance to cell-death-inducing therapies [187].

Mutations affecting genes involved in cell adhesion and the extracellular matrix, such as E-cadherin and MMPs (matrix metalloproteinases), promote the ability of cancer cells to invade adjacent tissues and metastasize to distant sites [65]. Loss of E-cadherin, whether due to mutations or epigenetic regulation, facilitates epithelial–mesenchymal transition, leading to tumor invasion and metastasis [188,189]. E-cadherin is encoded by the *CDH1* gene and is primarily responsible for forming adherent junctions between epithelial cells, which are crucial for maintaining tissue architecture and preventing cells from detaching and migrating [153]. The loss of E-cadherin disrupts cell–cell adhesion, allowing cancer cells to break free from the primary tumor and invade surrounding tissues. This detachment is a critical step in the initiation of metastasis, as cells gain the ability to migrate through the extracellular matrix and eventually enter the bloodstream or lymphatic system [189]. Consequently, the loss of E-cadherin is a hallmark of EMT, a process where epithelial cells lose their adhesive properties and acquire a more mesenchymal phenotype, enabling them to become more invasive and motile [153]. E-cadherin downregulation also contributes to tumor metastasis through the activation of signaling pathways that drive EMT, therefore promoting the expression of mesenchymal markers such as N-cadherin, vimentin, and fibronectin, which further enhance the migratory and invasive capabilities of cancer cells [190]. This switch from epithelial to mesenchymal characteristics enables cells to penetrate the basement membrane, a critical barrier to metastasis, and migrate toward distant sites, where they may form secondary tumors [191]. In OSCC, the loss of E-cadherin has been strongly correlated with poor prognosis and increased tumor aggressiveness, highlighting its importance in limiting metastatic potential [191]. Finally, the reduction in E-cadherin expression not only affects cellular adhesion, but also triggers signaling cascades, such as the Wnt/β-catenin pathway, which can further enhance tumor progression by promoting cell proliferation and survival [192]. This complex interplay between E-cadherin loss and EMT establishes a TME conducive to metastatic spread, making E-cadherin a critical factor in both the initiation and progression of metastasis in OSCC [193].

#### 4.3.4. Treatment Resistance

Genomic instability facilitates the emergence of cell subclones resistant to conventional therapies, such as chemotherapy and radiotherapy. This renders OSCC treatment more challenging, highlighting the need for more targeted and personalized therapeutic approaches [194]. For example, the genetic heterogeneity resulting from genomic instability can lead to the clonal selection of cells with mutations conferring resistance to specific chemotherapeutic agents, such as those targeting the EGFR pathway [91].

As previously discussed, miRNAs represent promising therapeutic targets in the treatment of OSCC [217,218]. Indeed, restoring the expression of tumor suppressor miRNAs or inhibiting oncomirs can potentially reverse the malignant phenotype of cancer cells [219]. Therapeutic strategies include the use of miRNA mimics (agomirs) to restore the function of suppressed miRNAs or miRNA inhibitors (antagomirs) to block the function of overexpressed miRNAs [220]. Preclinical studies have demonstrated that miR-21 inhibitors can efficiently restore the expression of various tumor suppressor genes and inhibit cell proliferation in OSCC models [221]. Gao et al. (2017) reported that the administration of miR-21 antagomirs resulted in significant tumor growth reduction in xenograft models [164]. The reintroduction of miR-34a through agomirs has shown significant antitumor effects, inducing apoptosis and inhibiting cell proliferation in OSCC [222]. In line with these observations, Li et al. (2021) showed that treatment with miR-34a agomirs resulted in tumor growth inhibition in murine models and increased chemotherapy sensitivity [223].

## 5. Conclusions

The carcinogenesis of OSCC is complex, involving various molecular and cellular mechanisms. Mutations in a number of tumor suppressors or oncogenes, such as *TP53* and *RAS*, are common and play crucial roles in malignant transformation. In addition to genetic mutations, epigenetic alterations, such as the hypermethylation of tumor suppressor gene promoters (p16INK4a and MGMT) and histone modifications, also play significant roles in OSCC carcinogenesis. Genomic instability, characterized by point mutations, chromosomal rearrangements, deletions, insertions, and aneuploidy, also contributes to tumor heterogeneity and the adaptation of cancer cells to various environmental and therapeutic stresses.

Risk factors such as tobacco use, excessive alcohol consumption, and infection with high-risk human papillomavirus (HPV) strains, particularly HPV-16, are strongly associated with the development of OSCC [18]. Tobacco smoking and chewing contribute to the majority of OSCC cases, as the chemicals found in tobacco are known carcinogens that induce DNA damage and promote mutations in key oncogenes and tumor suppressor genes. Combined with heavy alcohol use, the risk of OSCC increases significantly due to the synergistic carcinogenic effects of alcohol’s breakdown products, such as acetaldehyde, which enhances the mutagenic potential of tobacco carcinogens [65].

Other environmental factors include poor oral hygiene, chronic irritation from ill-fitting dental prostheses or sharp teeth, and nutritional deficiencies such as low consumption of fruits and vegetables [223]. These deficiencies can result in a lack of protective antioxidants, increasing susceptibility to DNA damage. Moreover, exposure to occupational hazards like asbestos and wood dust has been implicated in the development of OSCC, particularly in individuals working in environments where these materials are prevalent.

Additionally, genetic predispositions and immunosuppression, whether due to HIV infection or immunosuppressive therapies, are emerging as important risk factors. Immunocompromised patients are at a heightened risk of developing aggressive forms of OSCC due to their weakened ability to clear precancerous or virally infected cells [223].

Betel quid chewing, a practice common in parts of Asia, is another significant risk factor for OSCC. This habit, often combined with tobacco or areca nut, has been strongly associated with oral submucous fibrosis, a precancerous condition that can lead to OSCC. Ultraviolet (UV) light exposure has also been linked to the development of lip squamous cell carcinoma, a subtype of OSCC, particularly in outdoor workers [65]. A better understanding of the etiology of OSCC is essential for developing more effective and personalized therapeutic strategies to improve clinical outcomes for patients.

## Data Availability

Not applicable.
